# Gender equality and leadership in radiation oncology research: a plea to women to come forward

**DOI:** 10.1259/bjr.20230167

**Published:** 2023-08-15

**Authors:** Corinne Faivre-Finn

**Affiliations:** 1 Radiotherapy Related Research, University of Manchester & The Christie NHS Fondation Trust, Manchester, UK

## Abstract

This commentary paper describes a glass ceiling in the field of academia and specifically in radiation oncology research. Evidence from the literature and personal views are presented describing some of the issues leading to underrepresentation of women in academic leadership roles. The values and drivers for women in academia are discussed. Finally, a plea is made to women to come forward and consider leadership position and to academic institutions and funders of research to reconsider the traditional metrics of academic success.

We live in an era where gender equality is promoted by governments and employers. Women generally outperform men in educational attainment in the western world and have excellent career prospects, including in medicine and academia. However, a glass ceiling preventing women from advancing beyond a certain level has been described repeatedly, particularly in the field of academia.^1,2^ Concrete examples include the dismal representation of women in leadership positions in medical specialty societies and on governing and editorial boards^3,4^ and lower chances of promotion to the rank of associate professor, full professor, or department chair.^5^ The lack of gender parity within leadership teams of health care organisations and universities has a negative impact on women’s academic advancement.^6^ In addition, the COVID pandemic has had a profound negative effect on women's academic and leadership positions and outputs as they devoted more time to family responsibilities and household work than their male counterparts.^7^

So, are there differences between genders with regards to the perceptions and experiences of academic careers leading to the underrepresentation of women in leading roles? This commentary article is an expression of personal views with supporting evidence from the literature. My experience and views may not apply to all women working in radiation oncology research and some men may recognise some of the challenges described in this paper.

“Is there really an issue about women’s position in academia?”, this question is often heard coming generally from men and occasionally from women. In 2016, I published a commentary paper in *Clinical Oncology* entitled “Breaking the Glass Ceiling for Women in Academic Clinical Oncology in the UK: A Personal View”.^7^ This paper described the disproportionately small number of female academics in Clinical Oncology in the UK (*n* = 6) and discussed the barriers and potential solutions in helping women break the glass ceiling.^8^ Six years on, it is disappointing that although half of the clinical oncology workforce in the UK is female, the number of women holding a university chair has not changed.^9^ A number of societies and reports have since examined the question of gender equality in oncology and academia with similar conclusions on the underrepresentation of women, with little evidence of narrowing of the gap in the last three decades.^5^ In the USA, where women represent at least half of all medical school students, only 30% of applicants to radiation oncology training programs are female. The gender disparity continues to widen as women progress in their careers with only 17% of women holding leadership academic positions and 12% holding academic chairs in radiation oncology.^10^

In Europe, the European Society of Medical Oncology ‘Women for Oncology’ initiative should be applauded for its commendable mission statement of raising awareness and promoting equal access to career-development opportunities for female oncologists.^11^ A report from this group also highlighted the underrepresentation of women in leadership roles, members of boards or presidents of national and international oncology societies and as invited speakers at oncology congresses.^12^ Other practical initiatives include the provision of overall strategies for advancing women in leadership^6^ and resources available to support gender equity specifically in the field of radiation oncology.^13^ Policies from the most gender-equal countries are also useful resources for potential application within radiation oncology.^14^

In order to address this broken pipeline, an important consideration is understanding the values and drivers for women in the academic world. Although many studies have evaluated the representation and barriers to female leadership in academia, few have attempted to glean such understanding.^15^ My opinion is based upon years of observation of the academic world. Most women I have come across value the quality and impact of their work as well as relationships and collaborations. They also tend to overvalue expertise meaning that they focus more on the job delivery than their career and the associated politics. Such traits are engrained in our society and our upbringings, including the overwhelming self-doubt characteristic of the imposter syndrome that tends to affect women more than men. Women are generally more self-aware than men about work–life balance, choosing not to take on multiple roles associated with unrealistic expectations and a high risk of under delivery. Women increasingly reject the classical model of professional success designed over decades in many businesses and academic organisations by and for men. Many are keen to redefine success criteria to include values such as meaning, impact, ethics, flexibility, collaboration, collegiality, and greater permeability between professional and personal lives. Furthermore, it is also important to acknowledge that some women in medicine and academia make a conscious choice not to climb the career ladder and that should be respected.

Rather than weaknesses, such values and traits mean that women can often make excellent leaders as they are good at balancing professional and personal leadership skills. This is evidenced by women getting higher scores than men in the multisource feedback used during professional appraisals. Furthermore, in the business world, it has been demonstrated that gender-diversity leads to improved productivity, performance, innovation, as well as employee retention and satisfaction.^16^

Therefore, I make a plea to women who have an interest in academic medicine, please come forward and consider leadership positions. Evidence shows that your involvement will lead to higher quality research,^17^ promote a cooperative work style, improve interdisciplinary and team science as well as the diversity and inclusivity of research.^18^ Since the impact of your work and collegiality is an important value for you, taking up a leadership position is one the best ways to have a positive influence on the profession and to encourage women to follow your path. Furthermore, please consider taking on mentorship and importantly active sponsorship and advocacy roles for young women starting in the profession to help in facilitating their careers and providing a role model for them. Finally, my plea is also to the universities, NHS institutions and funders of healthcare research to reconsider the traditional and frankly dated measures of academic success, which include grant income, number of first and last author publications and the H-Index (an index capturing research output based on the total number of publications and the total number of citations). A new model embracing metrics that celebrate impactful and collaborative work, training and mentorship is more likely to be attractive, not only to women, but also to the new generation of radiation oncology researchers. Such a model has been recently initiated in the Netherlands. The Dutch Recognition & Rewards programme is a collaboration between universities, university medical centres, research institutes and research funders aiming to diversify and vitalise career paths, achieve a balance between individuals and the collective, stimulate open science and academic leadership and focus on quality and impact.^19^ I sincerely hope that other countries will take notice of this programme and consider similar initiatives in order to modernise and sustain academic activities in oncology and beyond.

*To help the reader understand my background and my motivation to discuss gender equality and leadership issues in radiation oncology research, I thought I would conclude this commentary paper with a series of facts and disclosures about myself. I have been working as an NHS consultant in clinical oncology for 22 years and as a clinical academic for the last 10 years, becoming a Professor of Radiation Thoracic Oncology in 2015. I have led numerous academic clinical trials, been an author of international guidelines and have leadership roles in several international organisations such as the European Organisation for Research and Treatment of Cancer (EORTC) and the International Association for the Study of Lung Cancer (IASLC). I am married to a General Practitioner who is very supportive of my career. We have two sons who are now young adults. My oldest son was diagnosed with Attention Deficit Hyperactivity Disorder (ADHD) in his early teens which added some complexity to my personal and professional life. Now in my sixth decade, I am eager to share my experiences of a very rewarding and stimulating career, particularly with younger colleagues. In addition to highlighting the positives of a clinical academic career, it is also important to be honest and open about the challenges associated with such a role, particularly as a woman, most of which I have experienced first-hand. They include the conflict between clinical and academic responsibilities, high demands relating to research outputs and work–life balance issues. Some of these challenges can be addressed, e.g. through mentoring, peer support, good time-management, a healthy lifestyle, self-awareness, and self-care; others may require action beyond the control of the individual.*
